# Mitochondrial aldehyde dehydrogenase obliterates insulin resistance-induced cardiac dysfunction through deacetylation of PGC-1α

**DOI:** 10.18632/oncotarget.11977

**Published:** 2016-09-12

**Authors:** Nan Hu, Jun Ren, Yingmei Zhang

**Affiliations:** ^1^ Shanghai Institute of Cardiovascular Diseases, Zhongshan Hospital, Fudan University, Shanghai, China; ^2^ Center for Cardiovascular Research and Alternative Medicine, University of Wyoming College of Health Sciences, Laramie, WY, USA

**Keywords:** sucrose, insulin resistance, ALDH2, Sirt3, PGC-1, Pathology Section

## Abstract

Insulin resistance contributes to the high prevalence of type 2 diabetes mellitus, leading to cardiac anomalies. Emerging evidence depicts a pivotal role for mitochondrial injury in oxidative metabolism and insulin resistance. Mitochondrial aldehyde dehydrogenase (ALDH2) is one of metabolic enzymes detoxifying aldehydes although its role in insulin resistance remains elusive. This study was designed to evaluate the impact of ALDH2 overexpression on insulin resistance-induced myocardial damage and mechanisms involved with a focus on autophagy. Wild-type (WT) and transgenic mice overexpressing ALDH2 were fed sucrose or starch diet for 8 weeks and cardiac function and intracellular Ca^2+^ handling were assessed using echocardiographic and IonOptix systems. Western blot analysis was used to evaluate Akt, heme oxygenase-1 (HO-1), PGC-1α and Sirt-3. Our data revealed that sucrose intake provoked insulin resistance and compromised fractional shortening, cardiomyocyte function and intracellular Ca^2+^ handling (*p* < 0.05) along with unaltered cardiomyocyte size (*p* > 0.05), mitochondrial injury (elevated ROS generation, suppressed NAD+ and aconitase activity, *p* < 0.05 for all), the effect of which was ablated by ALDH2. *In vitro* incubation of the ALDH2 activator Alda-1, the Sirt3 activator oroxylin A and the histone acetyltransferase inhibitor CPTH2 rescued insulin resistance-induced changes in aconitase activity and cardiomyocyte function (*p* < 0.05). Inhibiting Sirt3 deacetylase using 5-amino-2-(4-aminophenyl) benzoxazole negated Alda-1-induced cardioprotective effects. Taken together, our data suggest that ALDH2 serves as an indispensable cardioprotective factor against insulin resistance-induced cardiomyopathy with a mechanism possibly associated with facilitation of the Sirt3-dependent PGC-1α deacetylation.

## INTRODUCTION

Epidemiological studies estimate that the global prevalence of diabetes mellitus was 285 million among adults in 2010, and this number is anticipated to reach an alarming 439 million by 2030 [1, 2]. Among all contributing factors for diabetes, compromised insulin sensitivity is perhaps most crucial leading to the consequence of an overstimulation of pancreatic secretion of insulin and dampened glucose and fatty acid metabolism [3, 4]. Insulin resistance is associated with accumulation of reactive oxygen species (ROS), oxidative stress, onset and development of cardiac dysfunction possibly related to peroxidation of lipids and proteins, mitochondrial damage, and changes in excitation contraction coupling proteins [5]. Earlier work from our group and others has depicted that sucrose diet intake triggers whole body insulin resistance and cardiac dysfunction [6-8], which is in line with the clinical manifestations of cardiac dysfunction and increased mortality among patients with insulin resistance and type 2 diabetes [9]. Framingham Heart Study also correlates soft drink consumption with an increased incidence of metabolic risk, suggesting a close tie between carbonhydrate intake and metabolic diseases [10]. Nonetheless, neither the precise pathological mechanism nor effective clinical management is available for cardiac anomalies associated with sucrose diet-induced insulin resistance.

Recent evidence has indicated a key role for mitochondrial aldehyde dehydrogenase (ALDH2) in a wide array of heart diseases including ischemic heart disease [11-13], dilated cardiomyopathy [14], alcoholic cardiomyopathy [15, 16], diabetic cardiomyopathy [17, 18], and cardiac aging [19]. In addition, ALDH2 is reported to inhibit inflammatory response and regulate atherosclerotic plaque formation [20], which affects cardiac function indirectly. Up-to-date, the main cellular machineries behind ALDH2-offered cardiac benefits are perceived to be mediated through detoxification of toxic reactive aldehydes including 4-hydroxy-2-nonenal (4-HNE) and regulation of autophagy [11, 13, 15, 21-24]. Recent report also suggested a role for ALDH2 in the regulation of Sirt3 activation [25]. Sirt3 is one of sirtuin family proteins belonging to class III histone deacetylase (HDAC) localized predominantly in mitochondria. Sirt3 may serve as a mitochondrial stress sensor for the modulation of mitochondrial proteins governing metabolism [26, 27]. Given the important role for ALDH2 in cardiovascular diseases [17, 19, 21, 22, 28] and the high prevalence of cardiac dysfunction in insulin resistance [6-8], this study was designed to evaluate the impact of ALDH2 in cardiac function in the sucrose diet feeding-induced insulin resistance. In an effort to examine the possible mechanism of action involved in ALDH2-offered response to insulin resistance-associated cardiomyopathy, if any. Mitochondrial integrity was examined using aconitase activity, mitochondrial permeation pore opening (NAD+ levels) and mitochondrial biogenesis cofactor peroxisome proliferator-activated receptor-γ coactivator-1α (PGC-1α) as well as posttranslational modification of PGC-1α. Emerging evidence has revealed a likely role for the transcriptional coactivator PGC-1α in mitochondrial biosynthesis where reduced PGC-1α activity is associated with multiple glycolytic metabolisms in the heart [27, 29]. We also examined the levels of heme oxygenase-1 (HO-1), which is best known for its ability to regulate mitochondrial function and cardiac homeostasis in myocardial pathologies [30].

## RESULTS

### Echocardiographic properties of WT and ALDH2 mice challenged with sucrose

Neither sucrose feeding nor ALDH2 overexpression, or both, significantly affected body weight, heart weight and size (heart weight normalized to body weight), organ (liver and kidney) weight, heart rate, LV wall thickness, and LVEDD (*p* > 0.05 *vs.* starch group for all these indices). Sucrose diet feeding greatly enhanced LV ESD and suppressed fractional shortening (*p* < 0.05 *vs.* starch group for both indices), the effects of which were obliterated by ALDH2 transgene (*p* < 0.05 *vs.* sucrose group for both indices, Table 1). These data suggest that ALDH2 is capable of alleviating sucrose diet-induced myocardial dysfunction.

**Table 1:** General characteristics of WT and ALDH2 mice fed starch or sucrose diet

	WT-Starch	WT-Sucrose	ALDH2-Starch	ALDH2-Sucrose
Body Weight (g)	28.8 ± 0.6	30.9 ± 1.1	30.1 ± 0.8	31.2 ± 0.8
Heart Weight (mg)	130 ± 3	141 ± 3	127 ± 4	138 ± 4
Heart/Body Weight (mg/g)	4.53 ± 0.15	4.57 ± 0.13	4.24 ± 0.10	4.44 ± 0.05
Liver Weight (g)	1.19 ± 0.04	1.24 ± 0.04	1.21 ± 0.04	1.25 ± 0.05
Kidney Weight (g)	0.35 ± 0.02	0.37 ± 0.01	0.34 ± 0.01	0.34 ± 0.01
Heart Rate (bpm)	473 ± 12	467 ± 7	476 ± 12	474 ± 16
LV Wall Thickness (mm)	0.96 ± 0.08	0.95 ± 0.05	0.89 ± 0.08	0.93 ± 0.07
LVEDD (mm)	2.33 ± 0.16	2.49 ± 0.11	2.26 ± 0.13	2.25 ± 0.13
LVESD (mm)	1.04 ± 0.12	1.55 ± 0.19*	0.91 ± 0.11	0.95 ± 0.10#
Fractional Shortening (%)	54.6 ± 1.5	43.0 ± 2.1*	56.6 ± 1.5	53.3 ± 3.1#

LV, left ventricular; EDD, end-diastolic diameter; ESD, end-systolic diameter, Mean ± SEM, *n* = 6-7 mice per group,

* *p* < 0.05 *vs.* WT-Starch group,

# *p* < 0.05 *vs.* WT-Sucrose group.

### Effect of sucrose diet on energy expenditure and glucose tolerance in mice

**Figure 1:**
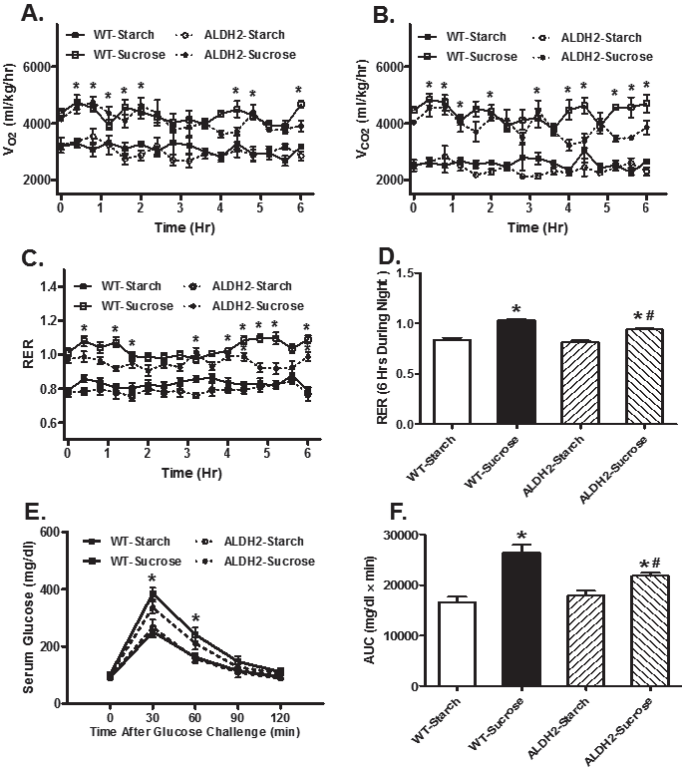
Effect of 8 weeks of sucrose diet (starch as control diet) intake on (**A**) oxygen consumption (VO_2_), (**B**) carbon dioxide production (VCO_2_), (**C**) real-time RER; (**D**) Pooled RER, (**E**) IPGTT curve and (**F**) area underneath the IPGTT curve in WT and ALDH2 transgenic mice. Mean ± SEM, *n* = 6- 8 mice per group, *p < 0.05 *vs.* WT-Starch group, #*p* < 0.05 *vs.* WT-Sucrose group.

Impact of insulin resistance and ALDH2 transgene on global metabolism was examined using the open-circuit indirect calorimetry. Our data revealed that diet-induced insulin resistance increased O_2_ consumption (VO_2_) (Figure 1A), CO_2_ production (Figure 1B) and respiratory exchange ratio (RER; Figure 1C & 1D) compared with starch group (*p* < 0.05 *vs.* starch group for all indices), favoring carbohydrate oxidation as a preferential energy source. Although ALDH2 transgene itself did not affect O_2_ consumption, CO_2_ production and RER (*p* > 0.05), it partially although significantly attenuated sucrose feeding-induced increase in RER (*p* < 0.05 *vs.* sucrose group) but not O_2_ consumption and CO_2_ production (*p* > 0.05 *vs.* sucrose group, Figure 1A-1D). Neither sucrose diet intake nor ALDH2 transgene, or both, significantly affacted total activity or heat production (data not shown). IPGTT test confirmed global insulin resistance following 8 weeks of sucrose diet feeding. Baseline glucose levels were unchanged by sucrose diet intake (*p* > 0.05 *vs.* starch group) although fasting blood glucose levels rose significantly at 30 and 60 min (*p* < 0.05 *vs.* starch group, Figure 1E), consistent with increased area under the curve (AUC) (*p* = 0.003 *vs.* starch group, Figure 1F) in sucrose diet group. ALDH2 transgene did not alter glucose tolerance in the starch-fed mice, although it partially but significantly attenuated sucrose feeding-induced increase in AUC (*p* = 0.024 *vs.* sucrose group, Figure 1F).

### Effect of sucrose diet intake and ALDH2 on cardiomyocyte cross-sectional area

**Figure 2:**
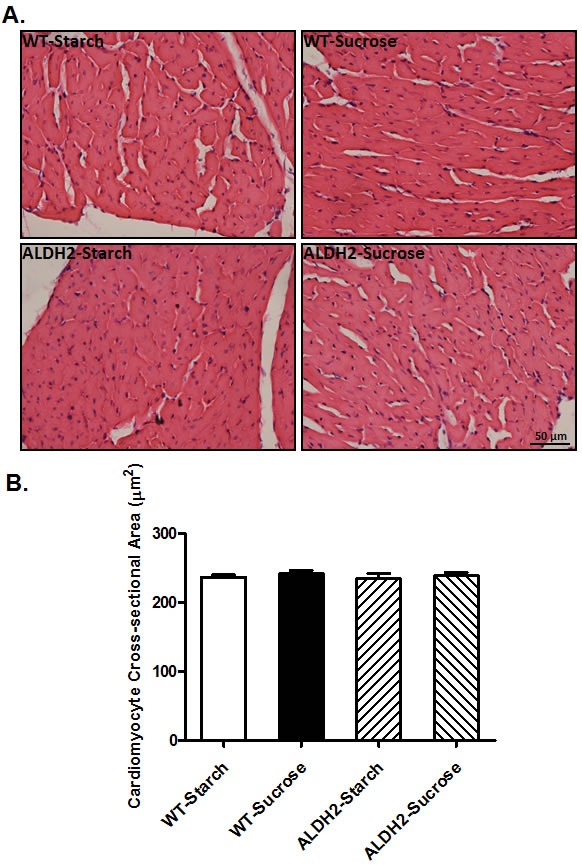
Effect of 8 weeks of sucrose diet (starch as control diet) intake on histological property in WT and ALDH2 transgenic mice **(A)** Representative H&E staining photomicrographs (original magnification 400×) of myocardial sections from respective groups; and **(B)** Pooled data of cardiomyocyte cross-sectional area. Mean ± SEM, *n* = 10-15 cells per mouse heart image from 5 mice per group, *p* > 0.05 among all groups.

Data from H&E staining revealed that neither sucrose diet nor ALDH2 overexpression, or both, affected cardiomyocyte cross-sectional area (*p* > 0.05 among all 4 groups, Figure 2A-2B), suggesting minimal impact of sucrose diet intake-induced insulin resistance or ALDH2 transgene on cardiac remodeling.

### Effect of sucrose diet intake and ALDH2 on cardiomyocyte contractile function and intracellular Ca^2+^handling

**Figure 3:**
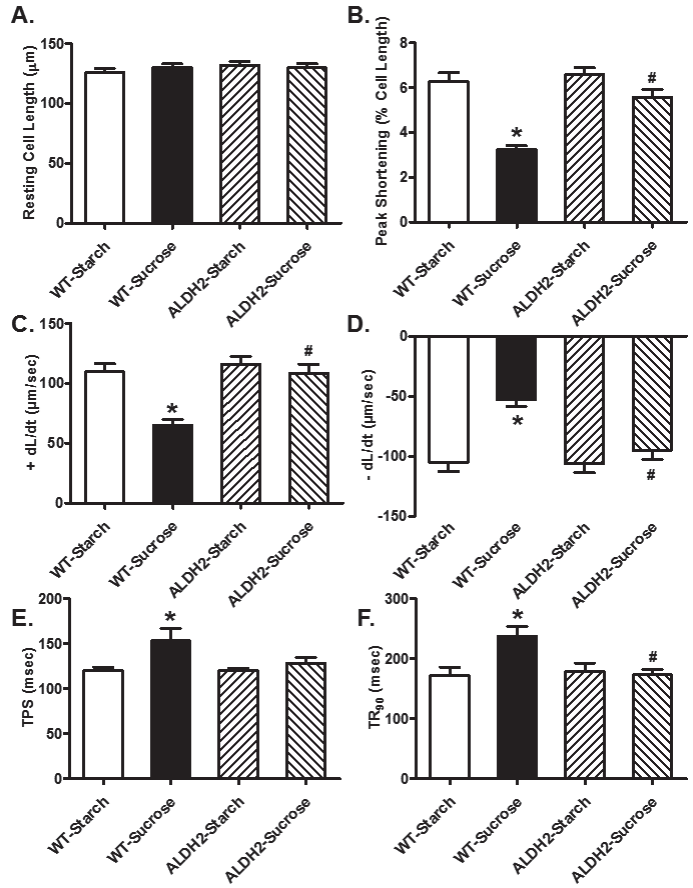
Effect of 8 weeks of sucrose diet (starch as control diet) intake on cardiomyocyte contractile function in WT and ALDH2 transgenic mice (**A)** Resting cell length; **B)** peak shortening; (**C)** maximal velocity of shortening (+ dL/dt); **(D)** maximal velocity of relengthening (-dL/dt); (**E)**, Time-to-peak shortening (TPS); and **(F)** Time-to-90% relengthening (TR_90_). Mean ± SEM, *n* = 70 - 74 cells from 3 mice per group, *p < 0.05 *vs.* WT-Starch group, # *p* < 0.05 *vs.* WT-Sucrose group.

**Figure 4:**
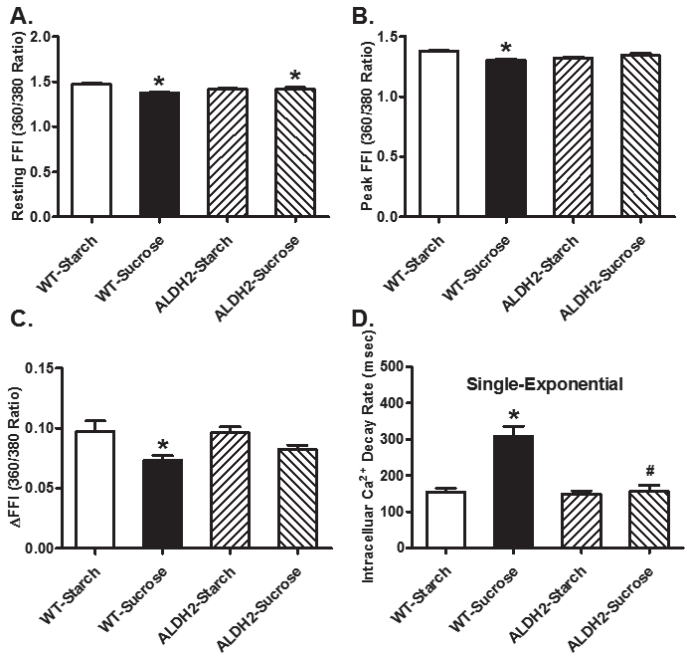
Effect of 8 weeks of sucrose diet (starch as control diet) intake on intracellular Ca^2+^ homeostasis in cardiomyocytes from WT and ALDH2 transgenic mice (**A)** Baseline fura-2 fluorescence intensity (FFI); (**B)** peak FFI; (**C)** change in FFI in response to electrical stimuli (ΔFFI) and **(D)** single exponential intracellular Ca^2+^ decay rate. Mean ± SEM, *n* = 60 cells from 3 mice per group, *p < 0.05 *vs.* WT-Starch group, # *p* < 0.05 *vs.* WT-Sucrose group.

Neither sucrose diet nor ALDH2 overtly affected phenotype of cardiomyocytes (data not shown) or resting cell length in cardiomyocytes. Sucrose diet feeding-induced insulin resistance resulted in a significant decrease in peak shortening (PS) and maximal velocities of shortening/ relengthening (± dL/dt) as well as prolonged TR_90_ (*p* < 0.05 *vs.* starch group for all these indices) without affecting TPS (*p* > 0.05 *vs.* starch group). Although ALDH2 transgene itself did not overtly affect these mechanical parameters (*p* > 0.05 *vs.* starch group), it significantly attenuated or abrogated (*p* < 0.05 *vs.* sucrose group) sucrose diet-induced mechanical changes (Figure 3). Data presented in Figure 4 displayed that sucrose diet significantly suppressed baseline and peak fura-2 fluorescence intensity (FFI), and electrically-stimulated rise in fura-2 fluorescence intensity (ΔFFI) as well as prolonging intracellular Ca^2+^ clearance (*p* < 0.001 *vs.* starch group for all these indices). Although ALDH2 itself did not affect these intracellular Ca^2+^ parameters (*p* > 0.05), it restored sucrose diet-induced changes in peak FFI, ΔFFI and intracellular Ca^2+^ decay rate (*p* < 0.001 *vs.* sucrose group).

### Effect of sucrose diet and ALDH2 feeding on total and phosphorylated Akt, HO-1, PGC1α, Sirt3 in mice

**Figure 5:**
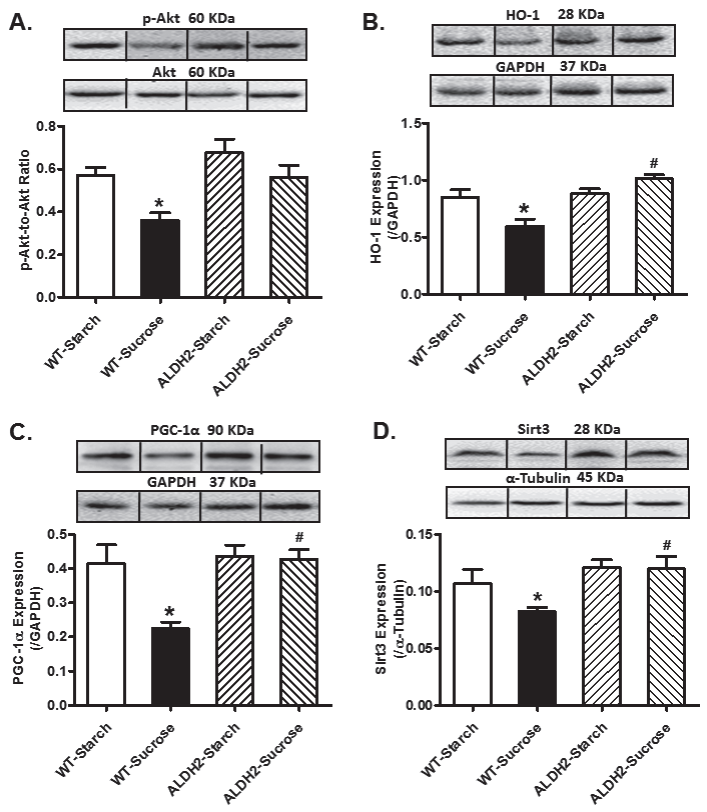
Effect of 8 weeks of sucrose diet (starch as control diet) intake on (**A**) phosphorylated levels of Akt; (**B**) protein level of HO-1, (**C**) protein level of PGC-1α; and (**D**) protein level of Sirt3 in WT and ALDH2 mice. Insets: Representative gel blots depicting expression of pan and phosphorylated Akt, HO-1, PGC-1α and Sirt3 using specific antibodies. GAPDH or α-tubulin was used as loading control; Mean ± SEM, *n* = 5 - 6 mice per group; *p < 0.05 *vs.* WT-Starch group, # *p* < 0.05 *vs.* WT-Sucrose group.

Our further analysis revealed that sucrose diet intake significantly suppressed Akt phosphorylation, as well as downregulated levels of HO-1, PGC-1α and Sirt3 (*p* < 0.05 *vs.* starch group for all these indices) without any overt effect on pan Akt levels. ALDH2 transgene itself failed to alter the levels of phosphorylated Akt, HO-1, PGC-1α and Sirt3 (*p* > 0.05 *vs.* starch group) although it significantly attenuated or abrogated (*p* < 0.05 *vs.* sucrose group) sucrose diet-induced changes in these protein markers (Figure 5).

### Effect of sucrose diet and ALDH2 on ROS production, aconitase activity and NAD+ activity

**Figure 6:**
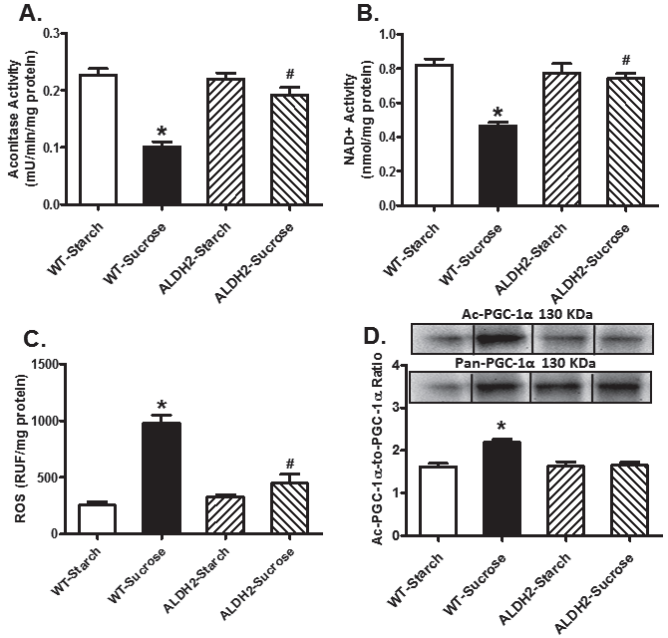
Effect of 8 weeks of sucrose diet (starch as control diet) intake on (**A**) aconitase activity; (**B**) NAD+ levels; (**C**) ROS accumulation; and (**D**) acetylated PGC-1α.protein levels in WT and ALDH2 mice. Insets: Representative gel blots depicting expression of pan and acetylated PGC-1α using specific antibodies. GAPDH was used as loading control; Mean ± SEM, *n* = 5 - 6 mice per group; *p < 0.05 *vs.* WT-Starch group, # *p* < 0.05 *vs.* WT-Sucrose group.

Assessment of mitochondrial function revealed that insulin resistance significantly decreased mitochondrial aconitase and NAD+ levels, suggesting mitochondrial injury. Aconitase is an iron sulfur enzyme located in citric acid cycle, and the mitochondrial aconitase activity is closely associated with oxidative stress related mitochondrial damage [21]. Although ALDH2 itself did not affect the activities of aconitase or NAD+ (*p* > 0.05 *vs.* starch group), it rectified insulin resistance-induced decrease in aconitase and NAD+ activities (*p* < 0.001 *vs.* sucrose group, Figure 6A-6B). Cardiomyocytes from WT and ALDH2 mice with or without sucrose diet intake were stained with DCF fluorescence dye prior to the assessment of tissue ROS levels. Our data revealed that insulin resistance overtly enhanced ROS generation (*p* < 0.001 vs. starch group), the effect of which was mitigated by ALDH2 (*p* < 0.001 *vs.* sucrose group). ALDH2 itself did not affect ROS production (*p* > 0.05, Figure 6C).

### Role of PGC1α acetylation in insulin resistance and ALDH2-induced cardiac responses

**Figure 7:**
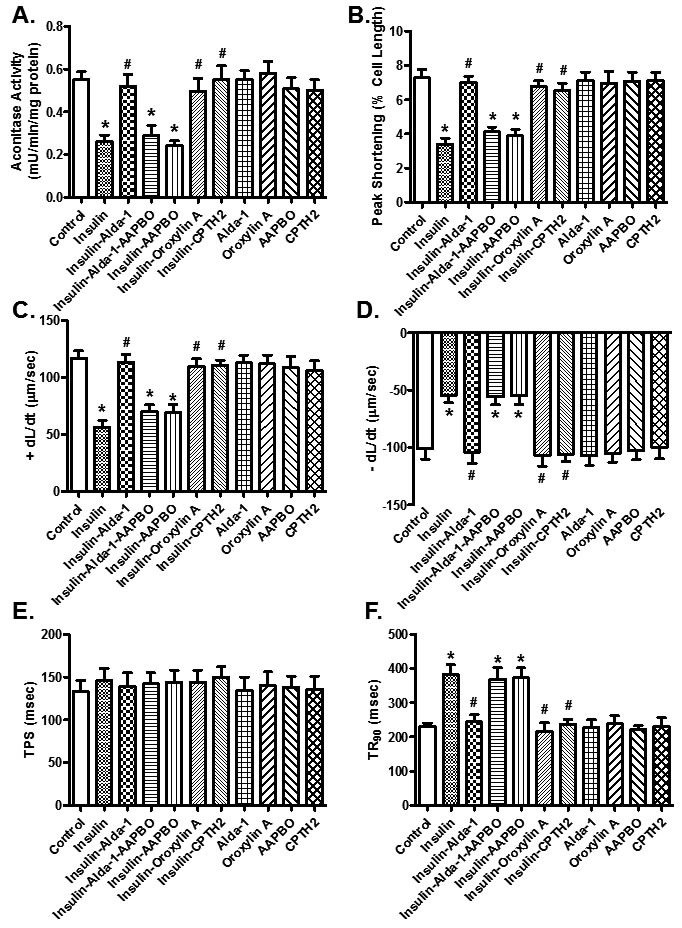
Assessment of aconitase activity and contractile function in cardiomyocytes cultured *in vitro* with high insulin (25 ng/ml, as a model for insulin resistance) for 2 hrs in the presence or absence of the ALDH2 activator Alda-1 (20 μM), the Sirt3 activator, Oroxylin A (100 μM) or the histone acetyltransferase inhibitor CPTH2 (200 μM). A cohort of Alda-1-treated high insulin cultured cardiomyocytes was co-incubated with the Sirt3 inhibitor 5-amino-2-(4-aminophenyl) benzoxazole (AAPBO, 100 μM) at the same time. **(A)** aconitase activity; **(B)** peak shortening, **(C)** maximal velocity of shortening (+ dL/dt), **(D)** maximal velocity of relengthening (-dL/dt); (**E)** time-to-peak shortening (TPS); and (**F)** time-to-90% relengthening (TR_90_). Mean ± SEM, *n* = 55 - 60 cells from 3 mice per group, *p < 0.05*vs.*control group, # *p* < 0.05 *vs.* insulin resistance group.

Given that Sirt3 is a crucial deacetylase regulating activity of PGC-1α [31], acetylated levels of PGC-1α were evaluated. Data shown in Figure 6D indicated insulin resistance promoted PGC-1α acetylation (*p* = 0.0213 *vs.* starch group), the effect of which was significantly attenuated by ALDH2 (*p* = 0.0213 *vs.* sucrose group) without any effect by ALDH2 transgene itself. To further discern a potential role of posttranslational modification of PGC-1α in ALDH2 transgene-offered protective effect against insulin resistance-induced cardiomyocyte anomalies, isolated murine cardiomyocytes from WT mice were pretreated with 25 ng/ml insulin for 2 hrs to establish insulin resistance *in vitro* [32, 33]. Assessment of aconitase activity and cardiomyocyte function revealed significantly decreased aconitase activity and cardiomyocyte function as manifested by reduced PS, ± dL/dt and prolonged TR_90_ in insulin resistant group (*p* < 0.05 *vs.* control group), in a manner reminiscent to *in vivo* insulin resistance elicited by sucrose diet feeding. Consistent with *in vivo* observation, co-incubation of the ALDH2 activator Alda-1 alleviated high insulin-induced mitochondrial and cardiomyocyte contractile anomalies (*p* < 0.05 *vs.* control group), the effect of which was cancelled off by Sirt3 inhibitor AAPBO (which promotes acetylation). The Sirt3 activator Oroxylin A and the histone acetyltransferase inhibitor CPTH2 obliterated (*p* < 0.05 *vs.* high insulin group) or overtly dampened insulin resistance-induced cardiomyocyte dysfunction and mitochondrial injury (aconitase activity) without eliciting any effects themselves. Moreover, the Sirt3 inhibitor AAPBO failed to produce additive effect to insulin resistance-induced cardiomyocyte contractile and aconitase anomalies (*p* > 0.05 between insulin and insulin-AAPBO groups for all indices, Figure 7).

## DISCUSSION

The salient findings of our study indicated that ALDH2 exerts a protective effect against insulin resistance-induced cardiac contractile dysfunction through preservation of mitochondrial integrity and Sirt3 function. Our findings indicated that sucrose diet intake-induced changes in myocardial contractile and intracellular Ca^2+^ properties in the absence of cardiac remodeling are closely associated with dampened phosphorylation of Akt and mitochondrial function. ALDH2 is likely to offer its protection by reversing insulin resistance-induced changes in mitochondrial integrity, Sirt3, and PGC-1α (pan/acetylated form). Furthermore, the possible involvement of Sirt3-mediated deacetylation of PGC-1α, in insulin resistance- and ALDH2-induced cardiac contractile and mitochondrial responses was substantiated by the pharmacological manipulation of Sirt3 acetylation and HAT. ALDH2-offered protection was recapitulated by the use of its agonist Alda-1, supporting the therapeutic potential of this enzyme in hyperinsulinemia. Interestingly, Alda-1-elicited beneficial effects against high insulin-induced cardiac dysfunction were effectively abolished by the specific Sirt3 deacetylation inhibitor AAPBO. Likewise, high insulin-elicited mitochondrial and functional anomalies were effectively reversed by the Sirt3 activator, Oroxylin A and the HAC inhibitor CPTH2. These findings support a pivotal role for the Sirt3 deacetylation in insulin resistance- and/or ALDH2-induced regulation of mitochondrial and contractile function in the heart. Taken together, these findings revealed that ALDH2 protects against insulin resistance-induced cardiac defect likely through a Sirt3-dependent deacetylation of PGC-1α.

The sucrose enriched dietary model offers an important means for cellular changes in the heart during early stages of development of type 2 diabetes and metabolic syndrome [6]. Data from the open-circuit calorimetry suggests presence of metabolic derangement following sucrose diet intake for 8 weeks. Balanced energy intake and expenditure is essential to a healthy energy homeostasis [34]. Our results did not notice any significant difference in total activity or heat production among all mouse groups, not favoring a role for energy expenditure in sucrose diet-induced metabolic and cardiac derangement. RER denotes the ratio between CO_2_ production and O_2_ consumption, suggesting the energetic substrate which is being oxidized. A higher RER ratio indicates increased oxidation of carbohydrate in reference to oxidation of fatty acid, whereas a lower RER denotes an increased oxidation of fat over carbohydrate [35]. In our hands, the RER value round 0.8 in the starch-fed mice suggests energy source from both fatty acid oxidation and carbohydrate oxidation [36]. With the high sucrose diet (68% of energy provided from sucrose which offers more carbohydrate relative to starch diet [37]), RER was significantly elevated to around 1 compared with the starch group, suggesting a more predominant role for carbohydrates as the energy source. Interestingly, ALDH2 transgene partially reversed sucrose diet-induced changes in RER, suggesting a role for the mitochondrial enzyme in governance of energetic substrate oxidation. However, ALDH2 transgene cannot fully alleviate sucrose diet-induced high carbohydrate oxidation as the predominant energy source.

Unfavorable changes in myocardial function are seen in sucrose-fed mice characterized by compromised contractility, prolonged diastolic duration intracellular Ca^2+^ mishandling [6, 37]. In our hands, sucrose diet-induced insulin resistance triggered myocardial contractile defects including enlarged LVESD, reduced fractional shortening, peak shortening, ± dL/dt and prolonged TR_90_ in the absence of overt change in cardiomyocyte cross-sectional area. These findings are somewhat in agreement with the previous notion of myopathic changes in chronic alcoholism-, insulin resistant- and diabetic cardiomyopathy [4, 6, 9, 15, 17, 18, 37-39]. Our data revealed interrupted intracellular Ca^2+^ homeostasis in response to sucrose diet intake manifested as depressed baseline, peak and electrically-stimulated rise in intracellular Ca^2+^, as well as prolonged intracellular Ca^2+^ decay, denoting an essential role of intracellular Ca^2+^ dysregulation in insulin resistance-induced cardiomyopathy. Perhaps the most intriguing observation from our study is that overexpression of ALDH2 alleviated insulin resistance-induced myocardial injury and mitochondrial defect. Data from our current study revealed that ALDH2 counteracts insulin resistance-induced cardiomyopathy (myocardial, cardiomyocyte function and intracellular Ca^2+^ handling) possibly in association with regulation of Sirt3 and mitochondrial integrity. Although ALDH2 transgene itself did not affect intracellular Ca^2+^ homeostasis, it unveiled a dampened release of intracellular Ca^2+^ and decrease in intracellular Ca^2+^ clearance in pre-diabetic myopathic changes. These findings suggest that ALDH2 plays a role in insulin resistance challenge-disturbed cardiac homeostasis. In accordance with cardiac dysfunction, comprised mitochondrial integrity was noted under insulin resistance (decreased aconitase levels, HO-1 levels, and NAD+ activity and elevated production of ROS). Through degradation of heme and generation of cytoprotective byproducts, HO-1 helps to maintain mitochondrial quality control by disengaging the positive feedback between oxidative stress and mitochondrial injury [40]. Consistent with its effect on cardiac contractile and intracellular Ca^2+^ properties in the face of sucrose diet intake, ALDH2 alleviated insulin resistance-induced ROS accumulation, as well as loss in HO-1 levels, aconitase activity and NAD+ levels. This was further supported by the findings from *in vitro* studies using pharmacological regulation of Sirt3 and ALDH2. In particular, Sirt3 activator effectively mitigated high insulin-induced cardiomyocyte and mitochondrial injury whereas the Sirt3 inhibitor cancelled off Alda-1-induced beneficial effect against high insulin.

In the pre-diabetic insulin resistance state, the accumulation of ROS is evident in hearts. ALDH2 is known to lower ROS levels in humans and animals [21] although the mitochondrial enzyme itself may not directly work as an antioxidant against ROS production. Interestingly, Sirt3 is well recognized as critical inhibitor to ROS. To elucidate possible mechanisms of action behind ALDH2 on sucrose diet-induced cardiac derangement, levels of Sirt3 and PGC-1α were examined with western blotting. Pre-diabetes are known to downregulate PGC-1α protein levels [41], thus we evaluated the effects of sucrose diet intake on Sirt3 and PGC-1α protein levels. In addition, since Sirt3 is the most important deacetylase that contributes to deacetylation of PGC-1α [31], we evaluated the acetylated lysine levels of this mitochondrial biogenesis marker that modulates mitochondrial dynamics and oxidative stress. Our immunoprecipitation data further revealed that insulin resistance promoted PGC-1 acetylation, the effect of which may be reversed by ALDH2. These data revealed that cardiac dysfunction under sucrose diet-induced insulin resistance is probably the result of decreased nuclear genes encoding mitochondrial proteins as a result of PGC-1α acetylation [42]. Our *in vitro* insulin resistant model confirmed that the benefit of Alda-1 may be nullified by the Sirt-3 inhibitor 5-amino-2-(4-aminophenyl)benzoxazole. In addition, direct activation of Sirt3 mimicked the Alda-1-offered protection against high insulin-induced cardiomyocyte contractile function and mitochondrial integrity. In our hands, acetylation of PGC-1α was noted in insulin resistant mouse hearts, which may contribute to its activity and thus mitochondrial function. Downregulated or acetylated PGC1α has been shown in failing hearts in numerous rodent models [43, 44]. Given that PGC-1α acetylation can be induced through activation of GCN5 acetyl transferase [45, 46], CPTH2 was employed to specifically inhibit HAT which modulates the GCN5 network [46]. As expected, CPTH2 offered beneficial effect against high insulin-induced cardiomyocyte contractile and mitochondrial injuries.

Experimental limitations: Although our current study has provided several lines of causal relationship among cardiac mechanical function, insulin resistance and PGC-1α acetylation, these results should not offer conclusive answers to the precise pathogenesis of cardiac defect in human insulin resistance. Use of more clinically relevant models of insulin resistance (such as defects in insulin receptor or post-receptor signaling components) should help to better understand the role of PGC-1α and its post-translational modification in the progression of insulin resistance-induced cardiomyopathy. Moreover, other post-translational modification modalities should not be discounted for regulation of mitochondrial biogenesis at this time.

**Figure 8:**
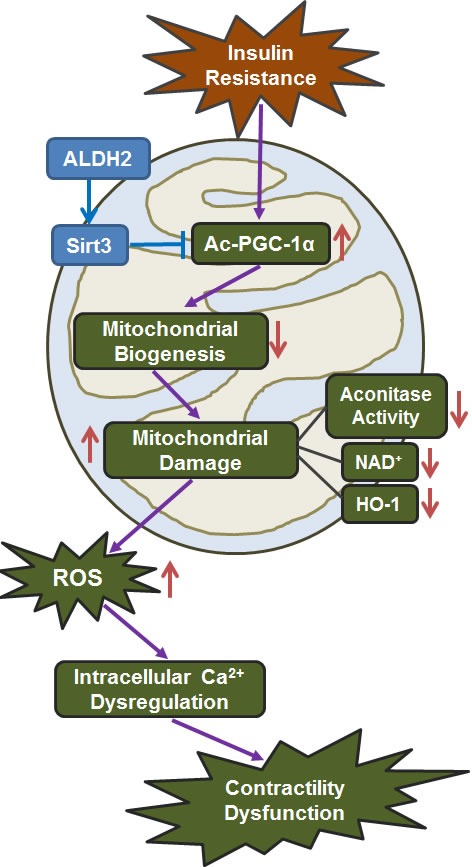
Schematic diagram depicting proposed mechanism for ALDH2-offered protection against insulin resistance-induced cardiac dysfunction. It is proposed that downregulation of acetylation of the mitochondrial biogenesis cofactor PGC-1α in response to insulin resistance challenge imposes suppression of mitochondrial function, *en route* to ROS production, intracellular Ca^2+^ derangement, and myocardial dysfunction. ALDH2 prevents insulin resistance-induced loss of PGC-1α function through Sirt3-mediated deacetylation to preserve mitochondrial integrity and cardiac function. Arrowheads refer to promoting subsequent response. ALDH2: mitochondrial aldehyde dehydrogenase; ROS: reactive oxygen species; Ac: acetylation.

In summary, findings from our present study have provided rather convincing evidence that ALDH2 rescues against cardiac anomalies including mechanical defect, intracellular Ca^2+^ dysregulation and mitochondrial injury through Sirt3-mediated deacetylation of mitochondrial biogenesis cofactor PGC-1α. Our data further revealed alleviated ROS production with ALDH2 overexpression under insulin resistance. A schematic diagram is provided here to summarize the proposed mechanism of action behind ALDH2-offered protection against insulin resistance-induced cardiac anomalies (Figure 8). Further study is warranted to unveil the mechanism behind ALDH2-mediated Sirt3 deacetylation in a more clinically relevant setting of insulin resistance-induced cardiomyopathy.

## MATERIALS AND METHODS

### Experimental animals, model of insulin resistance and intraperitoneal glucose tolerance tests (IPGTT)

Experimental procedures used here were approved by the Zhongshan Hospital Fudan University and University of Wyoming Institutional Animal Use and Care Committees. Insulin resistance was induced in Friend virus B (FVB) wild-type (WT) and ALDH2 transgenic mice. In brief, 3-month-old adult male mice were randomly divided into two groups to receive either starch diet or a sucrose diet (68% of energy from sucrose; Research Diets, D11725) for 8 weeks [6]. On the last day (23:00 hours) of diet treatment, food was removed and all mice were fasted for 12 hrs prior to IPGTT test. Mice were challenged with glucose (2 g/kg, ip) and blood was drawn immediately before and 30, 60, 90, and 120 min after glucose injection. Fasting blood glucose levels were assessed using an Accu-Chek glucose analyzer (Roche Diagnostics, Indianapolis, IN).

### Metabolic measurements

After 8 weeks of dietary treatment, mice were housed individually in metabolic cages, acclimated for 24 hrs to minimize the novelty effect, and then monitored for 24 hrs in 12-hour light/dark cycle by indirect open-circuit calorimetry (Oxymax System; Columbus Instruments, Columbus, OH) [47, 48]. Metabolic examination was performed over the course of 3 days while respective diet was maintained. Calibration of the calorimeter was performed at the beginning of each measurement day. Oxygen consumption and carbon dioxide production were measured by volume every 10 minutes. Respiratory exchange ratio (RER) was calculated as the ratio of VCO_2_/VO_2_. Energy expenditure was calculated as (3.815 + 1.232 × RER) × VO_2_ and normalized to body weight [49].

### Histological examination

Following anesthesia, hearts were excised and immediately placed in 10% neutral-buffered formalin at room temperature for 24 hrs after a brief rinse with PBS. The specimen were embedded in paraffin, cut in 5-μm sections and stained with hematoxylin and eosin (H&E). Cardiomyocyte cross-sectional areas were calculated on a digital microscope (x400) using the Image J (version1.34S) software [50].

### Echocardiographic assessment

Cardiac geometry and contractile function were evaluated in anesthetized (ketamine 80 mg/kg and xylazine 12 mg/kg, i.p.) mice using a 2-dimensional (2-D) guided M-mode echocardiography (Phillips Sonos 5500) equipped with a 15-6 MHz linear transducer (Phillips Medical Systems, Andover, MD). Adequate depth of anesthesia was monitored using toe reflex. The heart was imaged in the 2-D mode in the parasternal long-axis view with a depth setting of 2 cm. The M-mode cursor was positioned perpendicular to interventricular septum and posterior wall of left ventricle (LV) at the level of papillary muscles from the 2-D mode. The sweep speed was 100 mm/sec for the M-mode. Diastolic wall thickness, left ventricular (LV) end diastolic dimension (EDD) and LV end systolic dimension (ESD) were measured. All measurements were done from leading edge to leading edge in accordance with the Guidelines of the American Society of Echocardiography. LV fractional shortening was calculated as [(EDD-ESD)/EDD] × 100 [17].

### Cardiomyocyte isolation and cell mechanics

Hearts were removed rapidly from mice sedated with ketamine (80 mg/kg, ip) and xylazine (12 mg/kg, ip) and perfused with Krebs-Henseleit bicarbonate (KHB) solution consisting of (in mM) 118 NaCl, 4.7 KCl, 1.2 MgSO_4_, 1.2 KH2PO_4_, 25 NaHCO_3_, 10 HEPES, and 11.1 glucose. Hearts were digested with Liberase BlendzymeTH (Roche Diagnostics) for 15 min. After removal and mincing of the left ventricle, Ca^2+^ was added back to a final concentration of 1.25 mM. Cardiomyocytes with no spontaneous contractions and clear edges were used for shortening and Ca^2+^ cycling experiments. The IonOptix soft-edge system (IonOptix, Milton, MA) was employed to assess the mechanical properties of isolated myocytes. Myocytes were mounted on the stage of an Olympus IX-70 microscope in contractile buffer containing (in mM) 131 NaCl, 4 KCl, 1CaCl_2_,1 MgCl_2_, 10 glucose, and 10 HEPES. Myocytes were stimulated at0.5 Hz with cell shortening and relengthening evaluated using the following indices: peak shortening (PS), time to peak shortening (TPS), time to 90% relengthening (TR_90_), and maximal velocities of shortening/relengthening (± dL/dt) [51].

### Intracellular Ca^2+^ transients

A cohort of myocytes was loaded with fura-2/AM (0.5 μM) for 10 min, and fluorescence intensity was recorded with a dual-excitation fluorescence photomultiplier system (IonOptix). Myocytes were placed onto an Olympus IX-70 inverted microscope and imaged through a Fluor 40 oil objective. Cells were exposed to light emitted by a 75W lamp and passed through either a 360 or a 380 nm filter, while being stimulated to contract at 0.5 Hz. Fluorescence emissions were detected between 480 and 520 nm, and qualitative change in fura-2 fluorescence intensity (FFI) was inferred from the FFI ratio at the two wavelengths (360/380). Fluorescence decay time was measured as an indication of the intracellular Ca^2+^ clearing rate. Single exponential curve fit was used to calculate the intracellular Ca^2+^ decay constant [52].

### Intracellular fluorescence measurement of ROS

Intracellular ROS were measured by changes in fluorescence intensity resulting from intracellular probe oxidation [53]. Cardiomyocytes were loaded with 5-(6)-chloromethyl-2′,7′-dichlorodihydrofluorescein diacetate (CM-H_2_DCFDA, 1 μM, Molecular Probes, Eugene, OR) for 30 min at 37°C. Fluorescence intensity was measured at an excitation wavelength of 480 nm and an emission wavelength of 530 nm [53].

### *In vitro* insulin resistant model

Insulin was added to isolated cardiomyocytes to induce insulin resistance at a level of 25 ng/ml for 2 hrs [32, 33]. To assess the effect of ALDH2 activation and Sirt3 on insulin resistance-induced change in cardiomyocyte function, freshly isolated murine cardiomyocytes from WT mice were pretreated with the ALDH2 activator, Alda-1 (20 μM) [54], the Sirt3 activator, Oroxylin A (100 μM) [55], the Sirt3 inhibitor, 5-amino-2-(4-aminophenyl) benzoxazole (AAPBO, 100 μM) [56] or the histone acetyltransferase inhibitor CPTH2 (200 μM) [57] for 2 hrs prior to assessment of cardiomyocyte mechanical function.

### Aconitase activity

Mitochondria prepared from whole heart homogenate were resuspended in 0.2 mM sodium citrate. After determination of protein content, aconitase activity assay (Aconitase activity assay kit, Aconitase-340 assay™, Oxisresearch, Portland, OR) was performed according to manufacturer instructions with minor modifications. Briefly, mitochondrial sample (50 μl) was mixed in a 96-well plate with 50 μl trisodium citrate (substrate) in Tris-HCl pH 7.4,50 μl isocitrate dehydrogenase (enzyme) in Tris-HCl, and 50 μl NADP in Tris-HCl. After incubating for 15 min at 37°c with 50 rpm shaking, the absorbance was dynamically recorded at340 nm every min for 5 min with a spectramax 190 microplate spectrophotometer. During theassay, citrate is isomerized by aconitase into isocitrate and eventually α-ketoglutarate. Theaconitase-340 assay™ measures NADPH formation, a product of the oxidation of isocitrate to α-ketoglutarate. Tris-HCl buffer (pH 7.4) served as a blank. All results were normalized to respective protein content [58].

### Determination of NAD+

NAD+ was extracted from frozen ventricular tissues using perchloric acid. For these determinations, 30 mg of fresh frozen tissue was powdered in a mortar under liquid nitrogen and thoroughly mixed with 150 μl 0.6 M perchloric acid. The mixture was then homogenized, neutralized with 150 μl 3 M potassium hydroxide. NAD+ concentrations were determined fluorometrically in dilutions of the supernatant sample using alcohol dehydrogenase (Sigma-Aldrich, St. Louis, MO). Excitation was at 339 nm, and emission wavelength was at 460 nm in a spectrofluorimeter (Spectra MaxGeminiXS, Sunnyvale, CA) [58].

### Western blot analysis

Heart tissue was homogenized in a RIPA lysis buffer and centrifuged at 4°C for 20 min at 12,000xg. Supernatants were removed and protein concentration was measured using the Bradford assay. About 50 μg of equivalent protein samples were separated on a 7%, 10%, or 15% SDS-PAGE gel in a mini gel apparatus (Mini-PROTEAN II;Bio-Rad). Membranes were blocked with 5% milk in Tris-buffered saline/Tween 20 and incubated in anti-Sirt3, anti-PGC1α, anti-Akt, anti-phospho-Akt (pAkt), anti-HO-1, anti-GAPDH and anti-α-tubulin (Cell Signaling Technology, Beverly, MA) antibodies at the 1:1,000 dilution factor overnight at 4°C. Membranes were rinsed and incubated with horseradish peroxidase conjugated secondary antibodies and exposed using enzymatic chemiluminescence. The intensity of immunoblot bands was detected with a Bio-Rad Calibrated Densitometer (Model: GS-800). Images obtained by Western blot were analyzed using an ImageJ software (NIH) to quantify gel densities [51].

### Immunoprecipitation

Co-IP assay was performed following the protocol of the Co-IP kit (Pierce). Briefly, 50 μg of purified IR and PGC-1 antibodies were immobilized with coupling resin. Protein extracts (500 μg) were incubated with antibody-coupled resin gently end-over-end mixing for 2 hours at room temperature. The resin was washed, and the protein complexes bound to the antibody were eluted with 50 μl of elution buffer. The eluted protein was boiled and separated by 10% SDS-PAGE, transferred to a nitrocellulose membrane, and incubated with anti-Acetylated Lysine antibody. Antibody binding was detected using the enhanced chemiluminescence. The film was scanned and the intensity of immunoblotting bands was detected with a Bio-Rad Calibrated Densitometer (model GS-800) [38].

### Statistical analysis

Data were Mean ± SEM. Statistical comparison was performed by a 1-way ANOVA followed by the Tukey *post hoc* test. Significance was set at *p* < 0.05.
